# Role of Kupffer cells in the progression of CRC liver metastases after the first stage of ALPPS

**DOI:** 10.1038/s41598-018-26082-4

**Published:** 2018-05-24

**Authors:** Rocio García-Pérez, Joana Ferrer Fábrega, Aranzazu Varona-Bosque, Carlos Manuel Martínez, Beatriz Revilla-Nuin, Laia Cabellos, Romina Pena, Ramón Vilana, Carolina Gonzalez-Abós, Juan Carlos García-Valdecasas, José Fuster Obregón

**Affiliations:** 1 Liver surgery and Transplantation Unit, Department of surgery, ICMDM, Hospital Clinic, Barcelona, Spain; 2 BCLC Group, Liver Unit, Hospital Clinic, University of Barcelona, Barcelona, Spain; 3 Pathology Unit, IMIB Arrixaca, Murcia, Spain; 4 IDIBAPS, Barcelona, Spain; 5 Department of Radiology, CDI, Hospital Clinic, Barcelona, Spain; 6 CIBERehd, Barcelona, Spain

## Abstract

Associated liver partition and portal vein ligation for staged hepatectomy (ALPPS) has been suggested as a potential therapy for extensive bilobar liver tumors, although in some circumstances this technique may induce tumor progression, a fact still not well studied. Our aim was to study tumor hepatic progression induced by the first step of ALPPS in a WAG/Rij rat syngenic model of metastatic colorectal carcinoma by subcapsular CC531 cell line inoculation. ALPPS induced: tumor progression on deportalized lobe and metastases; expression of hepatic vasculogenic factors (HIF1-α and VEGF); and a dramatic increase of Kupffer cells (KCs) and tumor-associated macrophages (TAMs). Interestingly, KCs expressed COX-2 (M1 polarization), while TAMs expressed mainly arginase-1 (M2 polarization). ALPPS also induced a decrease of tumor-infiltrating lymphocytes and an increase of intrahepatic T lymphocytes. Thus, ALPPS technique seems to induce a hypoxic environment, which enhances hepatic HIF1-α and VEGF expression and may promote KCs and TAMs polarization. Consequently, the regenerative stimulus seems to be driven by a pro-inflammatory and hypoxic environment, in which M1 intrahepatic macrophages expressing COX-2 and T-Lymphocytes play a key role, facts which may be related with the tumor progression observed.

## Introduction

The liver is the organ most commonly affected by distant metastases from colorectal cancer (CRC)^1^. The development of colorectal cancer metastases is a multi-step process involving a complex interaction of several extrinsic factors which appear to play a critical role in tumor development^2^.

Liver resection is currently considered as the only potential curative therapy for CRC metastatic disease^3^. In place of standard techniques used for multiple bilobar CRC metastases, associated liver partition and portal vein ligation (ALPPS) has been proposed as a potential therapeutic surgical approach to induce greater hypertrophy of the future liver remnant (FLR) in a shorter period of time^3,4^. The biological reasons for such rapid hypertrophy are still unclear. However, there is mounting evidence that emphasizes the role of hypoxia-induced vasculogenesis and inflammation in such regenerative processes^5,6^, and its effect on tumor recurrence brings into question its application in surgical practice^4,7^.

In spite of experimental studies proving early tumor recurrence on hepatectomy and hypoxia-related portal vein occlusion models^8–11^, there are, as yet, no studies regarding the kinetics of hepatic CRC metastases following the ALPPS procedure. Thus, the aim of this study was to analyze, in an experimental rat model, the kinetics of tumor progression of CRC hepatic metastases induced by ALPPS, focusing mainly on the immune cell response induced by this technique (Kupffer cells (KCs), tumor-associated macrophages (TAMs), and T-lymphocytes).

## Results

### Macroscopic liver pathology and signs of extrahepatic metastatic disease

**Figure 1:**
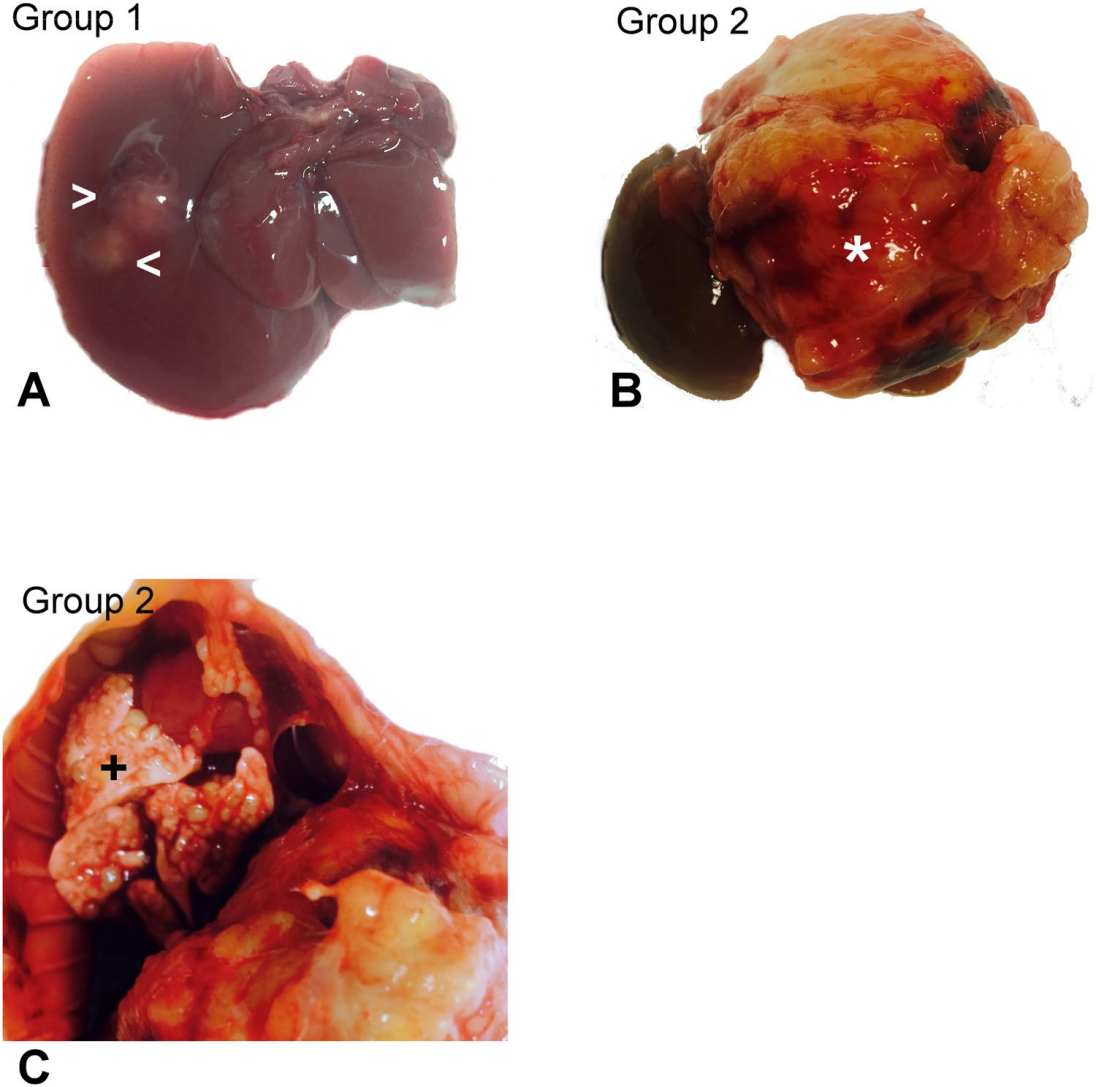
Representative images of progression of tumor disease in animals with subcapsular inoculation of syngenic CCR cell culture. In animals from group 1 (**A**), tumor cells formed a well-demarcated nodule protruding liver surface (head arrows). In contrast, groups in which an ALPPS procedure was performed, in group 2 (**B**) an obvious intrahepatic tumor progression was observed (asterisk). Additionally, signs of metastatic disease could also be evidenced in the lungs of one animal (**C**,+).

The livers from group 1 showed a well-circumscribed nodule in the area of inoculation on the affected lobe (Fig. 1A), without macroscopic signs of extrahepatic metastases. In contrast, the livers of animals from group 2 showed clear macroscopic signs of intrahepatic tumor progression on deportalized lobes (Fig. 1B). Liver weights showed significant differences between non operated and operated groups (Table 1, p = 0.002). Additionally, one animal from group 2 showed macroscopic signs of lung metastases (Fig. 1C).

**Table 1:** Histopathologic paramaters of tumor cells and presence of non-parenchymal hepatic cells on liver and inoculated tumor from groups.

Group	Liver Weight (GR.)	Tumor Proliferative Index (PI) (%/HPF)	Flr Kupffer Cells (Kc (cells/HPF))	Tumor-Associated Macrophages (TAMs) (cells/HPF)	Hepatic Infiltrating T-Lymphocytes (cells/HPF)	Tumor-Infiltrating T-Lymphocytes (cells/HPF)
1	9.93 ± 0.50	38.1 ± 6.59%	26.03 ± 3.73	12.20 ± 2.25	2.95 ± 0.76	22.87 ± 6.90
2	30.55 ± 7.84	37.25 ± 3.98%	80.53 ± 6.00	27.89 ± 4.35	9.55 ± 2.37	5.35 ± 1.94
3	11.54 ± 0.91	N/A	30.85 ± 4.84	N/A	4.45 ± 0.99	N/A
4	7.71 ± 0.62	N/A	18.35 ± 2.66	N/A	3.23 ± 1.09	N/A

HPF: high-power-field.

### Histopathology

**Figure 2:**
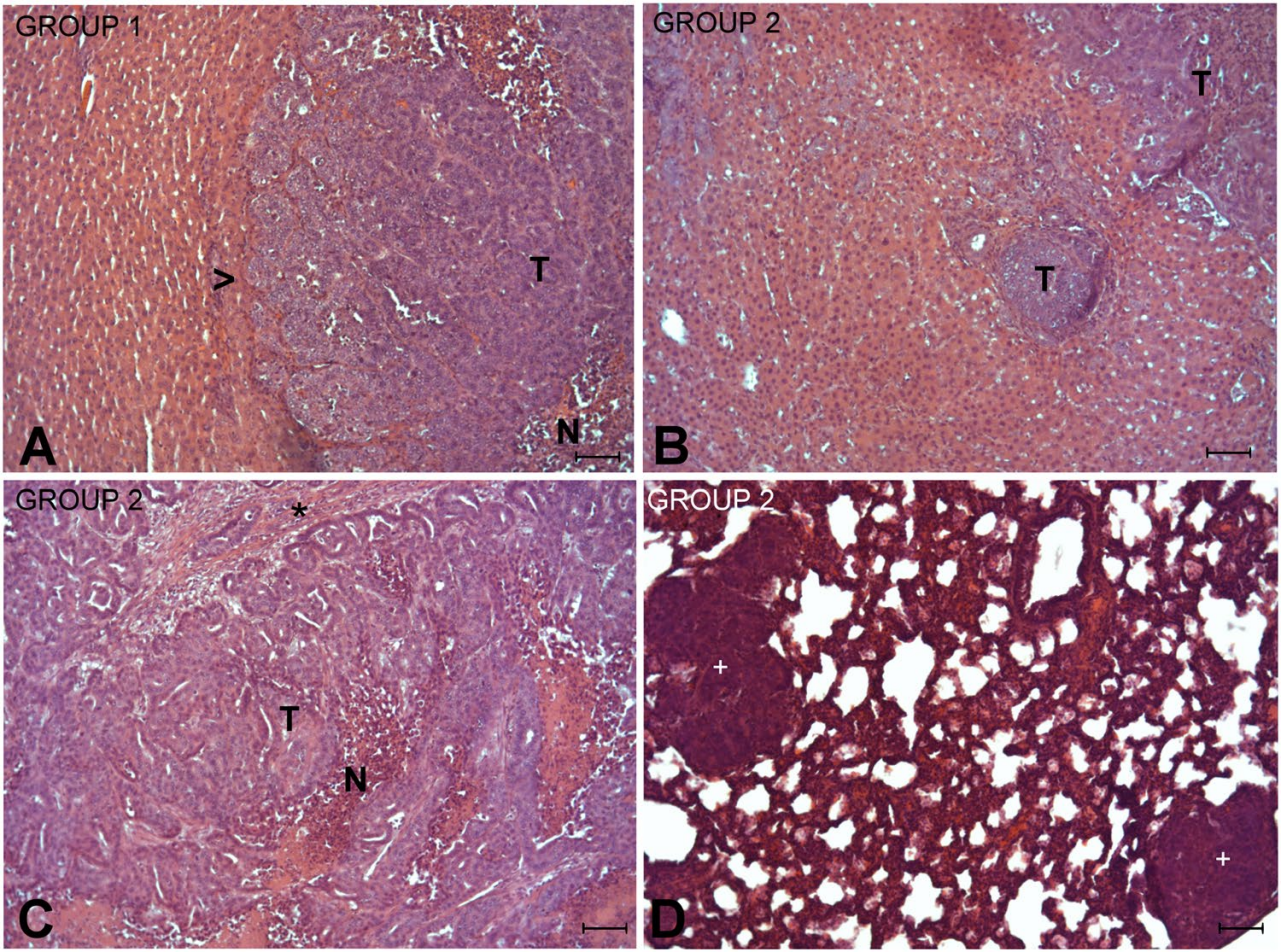
Representative images of the main histopathology events in the livers from animals of group 1 (**A**), and FLR (**B**), deportalized lobes (**C**) and lungs (**D**) from animals of group 2. Subcapsular inoculation of tumoral CCR cells on group 1 (inoculation and no operated, A) induced a nodule of tumor cells (T) independently of the lobe involved. The nodule is well circumscribed, with an expansive growing pattern which induce atrophy of adjacent hepatocytes (head arrows) and scattered areas of necrosis (N). In group 2, the first ALPPS step led to a tumor progression either in FLR of 3 animals (**B**, T) and in the deportalized lobe (**C**), with a villous pattern, areas of necrosis (N) and minimal stroma (asterisk). Additionally, lung metastasis (**D**, + ) could be also observed in some animals from this group. Hematoxylin and eosin stain. Scale bar: 100 μm.

The histopathology of animals from group 1 (Fig. 2A) revealed a well-circumscribed tumor nodule with an expansive growing pattern, and occasionally areas of central necrosis, without signs of tumor progression. The healthy liver from operated groups (groups 2 and 3) showed slight sinusoid dilation, biliary hyperplasia and scattered periportal mitotic figures. Interestingly, in animals from group 2 (Fig. 2B,C) not only was a clear tumor progression observed in deportalized lobes, but there was also tumor progression in FLR of 3 animals. No histopathologic findings were identified in livers from animals from group 4. Additionally, examination of the lungs confirmed the presence of metastases in 2 animals from group 2 (Fig. 2D).

The tumor proliferative index (PI) analysis showed no significant differences between non-operated (group 1) and operated group (group 2) (Supplementary Fig. 1A,C and Table 1). In contrast, with β-catenin expression, while low numbers of tumor cells from group 1 expressed a weak membrane staining pattern (Supplementary Table S1, Suppl. Fig. 1B), moderate numbers of tumor cells (15–30%) from group 2 expressed a strong cytoplasmic and/or nuclear staining (Supplementary Table S1, Suppl. Fig. 1D).

### Characterization of non-parenchymal hepatic cells

**Figure 3:**
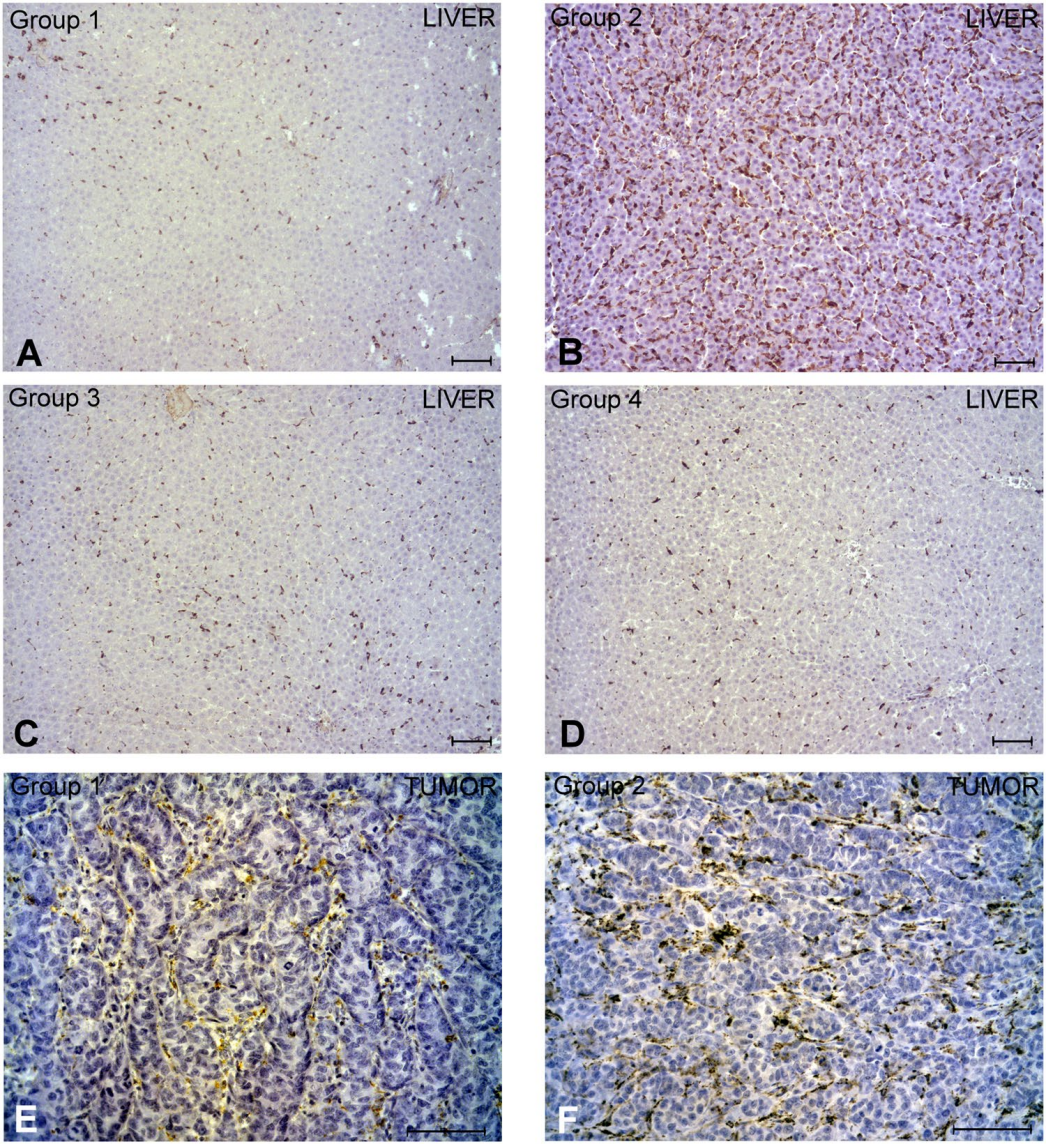
Representative images of Kupffer cells (KCs) in hepatic tissue (**A**,**B**,**C**,**D**) and tumor-associate macrophages (TAMs) (**E**,**F**) from group with only inoculation of CCR cell line without surgery (group 1, **A)**, inoculation of CCR cells on deportalized lobe and first step of ALPPS (group 2, **B**,**E**,**F**), first ALPPS step only (group 3, **C**) and only laparotomy (sham) group (group 4, **D**). In group 2 (**B**) there was a dramatically increase of KCs, not only in comparison with group 3 (**C**), but also with non-operated groups (**A**,**D**). Regarding TAMs, there was also a significant increase in this cell subpopulation in group 2 (**F**) in comparison with group 1 (**E**). ABC Anti-CD68 stain. Scale bar: 100 μm.

Tumor cell inoculation (group 1) induced a significant increase of hepatic KCs in comparison with the sham group (group 4) (Table 1, p < 0.0001, Fig. 3A,D). Similarly, the first step of ALPPS (groups 2 and 3) induced a significant increase of parenchymal KCs in comparison with non-operated groups (groups 1 and 4, Table 1, p < 0.001, Fig. 3A–D), especially in group 2 in which the dramatic increase of KCs was significant in comparison with all other groups (Table 1 p < 0.0001). Regarding TAMs, the liver surgery (group 2) exhibited a significant increase in their numbers in comparison with the inoculated and non-operated group (Table 1, p < 0.0001, Fig. 3E,F).

Regarding COX-2 expression on KCs, very few cells from group 1 were positive (Suppl. Table S1, Supplementary Fig. 2A), whereas all KCs from group 2 and 3 expressed COX-2 (Suppl. Table S1, Suppl. Fig. 2B,C). KCs of group 4 were all negative. KCs were negative for arginase-1 staining. Regarding TAMs, while very few arginase-1 or COX-2 positive cells could be observed in group 1 (Suppl. Table S1, Supplementary Fig. 3A,B), there was a high number of arginase-1 and low numbers of COX-2 positive cells from group 2 (Suppl. Table S1, Suppl. Fig. 3C,D).

The analysis of T-cell infiltrate on hepatic tissue (Table 1, Supplementary Fig. 4) revealed that inoculation without surgery did not increase T-cell infiltrate (Table 1, Supplementary Fig. 4A). When the first stage of ALPPS method is performed (group 3), there is a slight increase of T-cell infiltrate in comparison with the sham group (group 4) (p < 0.01) (Table 1), and a marked increase when tumor cell inoculation is performed (group 2) (p < 0.001) (Table 1, Suppl. Fig. 4C). Regarding TILs, the elevated quantity of infiltrating CD3+ T-cells from group 1 (Table 1, Suppl. Fig. 4B) was significantly higher than group 2 (p < 0.001) (Table 1, Suppl. Fig. 4D).

### Expression of vasculogenic factors (HIF1-α and VEGF)

Regarding expression of vasculogenic factors, hepatocytes from group 1 were negative for HIF1-α (Suppl. Table S1), but when the first stage of ALPPS is performed, 5–15% of hepatocytes expressed HIF1-α and 15–30% expressed VEGF (Suppl. Table S1). Regarding expression of vasculogenic factors of tumor, cells of group 1 were negative for HIF1-α expression and 5–15% were positive for VEGF (Suppl. Table S1, Supplementary Fig. 5A,B). Interestingly, tumor cells from group 2 were also negative for HIF1-α yet were strongly positive for VEGF expression (Suppl. Table S1, Suppl. Fig. 5C,D).

## Discussion

ALPPS has arisen as a new technique to induce quicker liver regeneration in the FLR compared to classic techniques (PVE or PVL). Numerous variants on this technique have been published^12^ since the first study proposed by Schnitzbauer *et al*.^13^. When this technique was described, the main disadvantage was its high morbidity and mortality rate, characteristics that have been continuously reduced as experience in this field grows. Thus, there are reports that state up to 50% of overall survival and 13% of disease-free survival at 3 years after ALPPS procedure^14^. Moreover, there have been very recent reports developed in animal models that suggest that ALPPS does not affect tumor progression in the FLR during regenerative process in neither the first^15^ nor the second stage^16^. Nevertheless, early tumor recurrence (within the first 12 months after surgery) remains one of the main disadvantages of this technique^14,17^. Although there are several hypothesis that try to explain these results (e.g. failings in chemo-therapeutic treatment^15^), the reason for such progression is still unknown. Among the multiple regenerative stimuli driven by ALPPS, inflammatory signals may have an important role, especially Kupffer cells, as a source of pro-inflammatory mediators^6,18^. This seems to be a significant issue since recent reports point that the environment created by pro-inflammatory (M1) macrophages seems to promote colorectal cancer (CCR) progression^19^. As the influence of Kuppfer cell proliferation and response during ALPPS-induced regenerative stimulus on liver colorectal cancer metastatic progression remains to be elucidated, our purpose was to study such progression using a liver metastatic CRC experimental model in rats. This model describes the induction of CRC liver metastases by the subcapsular inoculation of tumor cells, which provides one of the most accurate *in-vivo* experimental models for the development of liver metastases in the site of inoculation within 1 month^10,11^.

The accuracy of the method was established on the basis of the analysis of animals from group 1, in which the tumor developed in the site of inoculation with no signs of extra hepatic progression or metastases.

As expected, ALPPS procedure without CRC cell inoculation induced an increase of Kupffer cells, a fact previously reported^6^. However, in metastatic disease progression, we observed that this increase rose dramatically. It has been established that the profile of cytokines secreted by KCs depends on the stage of liver regeneration process, from promitogenic promoters (pro-inflammatory or M1 profile) on the early start-up^20^, to proliferative inhibitors (pro-regenerative or M2 profile) on late phases^21^. Classically, hepatectomy models describe a M2 phenotype for KCs^22,23^ yet in our model, a strong expression of COX-2 was observed. As this enzyme is related with M1 polarization^23^, this appears to confirm previously reported accounts^6^ regarding pro-inflammatory factors during renegerative stimulus promoted by ALPPS. Moreover, the role of COX-2 in CCR progression has been well established^9,10^, so the expression of hepatic COX-2 observed in our model may have not only a role in liver regeneration, but also in metastatic progression. Additionally, a high expression of arginase-1 has been observed in TAMs from inoculated and operated groups; a hallmark of a M2 profile^24^. This is an important finding because recent evidence suggests that tumor hypoxia not only plays a key role in the phenotypic control of TAMs with M2 polarization, but that hypoxic TAMs release factors also contribute to tumor growth, cancer immunotolerance, angiogenesis and even chemotherapy resistance^25^. Thus, ALPPS proliferative stimulus may not only induce an M1 polarization of KCs in the healthy liver, but that hypoxic condition promoted by this technique could also contribute to a M2 polarization of TAMs, which may contribute to tumor progression.

The presence of TIL’s has been classically associated with a favorable prognosis in CRC^26^. In our model, we have observed a significant decrease of TIL’s but interestingly, an increase of T-lymphocytes in hepatic tissue may be related with the proinflammatory environment promoted by the regenerative stimulus. Although the reason needs to be investigated, it is known that certain T-cell subsets can regulate macrophages to promote tumor progression^23,26^, or to the development of a negative regulation of Th1 immune response^27^.

There is increasing evidence that confirms the tumorigenic role of vasculogenic factors secreted in response to hypoxia induced by portal vein occlusion techniques, a fact that it has been also observed in ALPPS^28^. In this sense, HIF1-α and VEGF expression seem to play a significant role in metastatic progression and are correlated with disease stage and poor prognosis^29^. It has been well- established that ALPPS can induce release of these factors in response to hypoperfusion of transected lobes and can play a part in proliferative stimulus and induce secretion of pro-inflammatory cytokines by KCs^30^. In our model, we observed liver HIF1α and VEGF expression, whereas tumor cells were positive only for VEGF. These results suggest that a hepatic peritumoral microenvironment may promote proliferation of tumor cells. These results invite us to consider that the carcinogenic progression in our model may be driven by a complex interaction between hypoxia factors and pro-inflammatory mediators, in which COX-2, mainly secreted by KCs, may have a key role.

In this report we decided to focus our studies on the deportalized lobe, which is able to partially preserve its functional capacity thanks to the maintenance of arterial flow^31^. The presence of distant (pulmonary) metastases in some animals in our model indicates that the possibility of distant metastases should be allowed for, even after performing the first stage of ALPPS.

Despite the facts of these important results, there are several limitations. Firstly, the anatomy of the rat liver is different to the human liver (four lobes vs. eight segments) and proliferative rate of e rat liver seems to be more pronounced than in the human model^32^. Secondly, human patients undergoing an ALPPS strategy have a complex and advanced oncological disease with a history of prolonged chemotherapy schemes^33^. Nevertheless, experimental models can represent a useful tool to study histological and molecular mechanisms involved in the physiology of liver regeneration induced by this technique, and to study if such mechanisms can affect tumor cells with or without complex clinical schemes. Thus, it is possible to establish an ALPPS model that induces a similar morphometric effects to its human counterpart^5,6^ and creates experimental models which mimic colorectal liver metastasis with partial hepatectomy^9^, portal vein ligation/embolization^11^ or even ALPPS techniques^10,15,16^, again with similar results to the human model.

In conclusion, according to our results, we can strongly suggest that there is a relation between the application of the surgical technique and the risk of tumor progression. More studies on the use of COX-2 inhibitors are needed to establish the exact effect of this enzyme on metastatic CRC hepatic progression promoted by ALPPS regenerative stimulus. Having said this, our results may support an interesting hypothesis regarding the carcinogenic progression induced by the regenerative stimulus caused by this technique, thus opening new perspectives about the use of COX-2 inhibitors (experimentally demonstrated^34^) to prevent such progression in order to maximize proliferative effects and minimize tumor progression risk.

## Methods

### Animals and Ethical Statement

All experiments were performed on male WAG/Rij 8-week old rats (weighing 250–300 gr., Harlan, Madrid, Spain). Prior to surgery, all animals were kept in a specific pathogen-free (SPF) environment at the University of Barcelona animal facilities in strict accordance with the protocol was approved by the Committee on the Ethics of Animal Experiments of the University of Barcelona (Permission Number: 98/15).

Cell lines and culture CC531 cell line, derived from a dimethyl hydrazine-induced WAG rat colon adenocarcinoma (CLS Cell Lines Service GmbH, Berlin, Germany) was maintained as adherent monolayers in complete RPMI 1640, with 2% L-glutamine, 10% heat-inactivated fetal bovine serum (FBS) and 2% penicillin/streptomycin, all purchased from Gibco (Grand Island, NY).

### Experimental design: surgical procedures and induction of liver tumors

**Figure 4:**
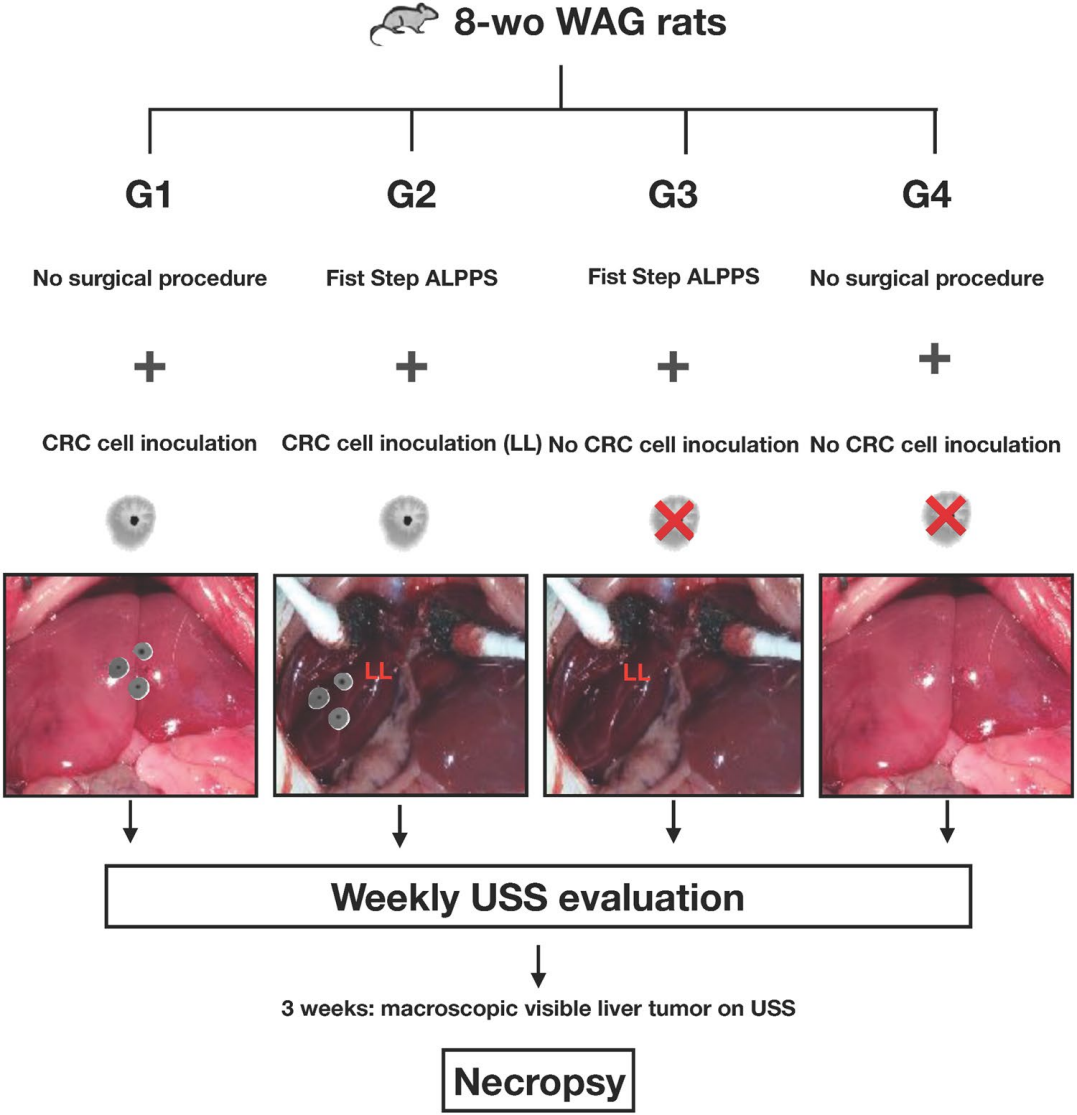
Timeline outlining experimental design and animal groups. All animals were examined by ultrasonography until the 3^rd^ week, where clear macroscopic signs of tumor were evidenced. LL: deportalized lobe.

A total of 24 rats were distributed in 4 groups (n = 6/each) as follows (Fig. 4):

- Group 1 (bilateral CRC inoculation without surgery): injection on the left and right portion of medium lobe.
- Group 2 (inoculation on the deportalized lobe and surgery): inoculation on the right lobe and right portion of the medium lobe (deportalized lobes).
- Group 3 (no inoculation and surgery): injection of phosphate-buffered saline (PBS) without cells on medium lobe.
- Group 4 (control group): only a midline laparotomy was performed in all animals of this group.

The induction of liver tumors was performed, after a midline laparotomy, by the subcapsular inoculation of 250 μl of sterile PBS containing one million CC531 tumor cells, using a 28 G needle in groups 1 and 2 in accordance with a previously described and standardized method^11^. Following the cell inoculation and in accordance with the methodology described previously^6^, portal vein ligation in right and medium right lobe and liver transection of the medium lobe were performed in groups 2 and 3 as shown in Fig. 4.

All the animals were then kept in a SPF and dark environment with daily observation until recovery. Weekly ultrasounds were routinely performed on the rats until the third week after surgery to ensure the correct implantation and location of the metastasis.

At this point, all animals were sacrificed followed by a regular necropsy procedure. Liver and tumor tissue samples were fixed in 4% buffered formalin (Panreac Quimica, Madrid, Spain) for 24 hours.

### Histopathology and immunohistochemistry

Fixed samples were processed, paraffin-embedded, 4μm-sectioned and stained with a standard hematoxylin and eosin (H&E). For tumor progression in inoculated groups, the following parameters were taken into account:

- Signs of macroscopic or microscopic pulmonary metastasis, liver weight, proliferative index (PI) (Ki-67, Master Diagnostica, Granada, Spain) and expression of β-catenin (Dako, Madrid, Spain).
- Characterization of non-parenchymal cells and other factors related with inflammation or immunoregulation: Kupffer cells (KCs) (anti-rat CD68, Millipore, Madrid, Spain), COX-2 (Thermo, Madrid, Spain), arginase-1 (Santa Cruz Biotech.) and T-CD3 lymphocytes (Dako).
- Vasculogenic factors: expression of VEGF (Abcam, Cambirge, UK) and HIF1-α (Santa Cruz Biotech., California, USA).

PI, KCs and T-CD3 lymphocyte quantities were estimated by averaging positive cell numbers in 10x high power fields (400x). All immunohistochemical procedures were performed by using an automated staining system (Dako), in accordance with the manufacturer protocols.

### Statistics

Statistical analysis was performed using a software package (Prism ver. 7.00, Graph Pad Inc., California, USA). All the numerical data are expressed as the ± standard deviation from the average value. Differences between groups for quantitative parameters were assessed by a Mann-Whitney non-parametric test on basis on the lack of a normal distribution according with Shapiro-Wilk normality test. A p-value of <0.05 was considered as significant. The size of the groups was estimated for a statistic power of 83.4% (GPower, Ver. 3.1.9.2), expecting medium-high differences between medians based on previous studies.

## Electronic supplementary material

Supplementary information
