# Supplementary material for: Role of Kupffer cells in the progression of CRC liver metastases after the first stage of ALPPS
Source: Sci Rep. 2018 May 24;8:8089. doi: 10.1038/s41598-018-26082-4 (PMC5967300; doi:10.1038/s41598-018-26082-4)

SUPPLEMENTARY INFORMATION

Role of Kupffer cells in the progression of CRC liver metastases after the first stage of ALPPS

Rocio García-Pérez[1], Joana Ferrer Fábrega[1,2], Aranzazu Varona-Bosque[1], Carlos Manuel Martínez[3], Beatriz Revilla-Nuin[3], Laia Cabellos[2,4], Romina Pena[1], Ramón Vilana[2,4,5], Carolina Gonzalez-Abós[1], Juan Carlos García-Valdecasas[1,4,6], José Fuster Obregón[1,2,4,6]

[1] Liver surgery and Transplantation Unit, Department of surgery, ICMDM, Hospital Clinic, Barcelona, Spain

[2] BCLC Group, Liver Unit, Hospital Clinic, University of Barcelona, Barcelona, Spain.

[3] Pathology Unit, IMIB Arrixaca, Murcia, Spain

[4] IDIBAPS, Barcelona, Spain

[5] Department of Radiology, CDI, Hospital Clinic, Barcelona, Spain

[6] CIBERehd, Barcelona, Spain

SUPPLEMENTARY FIGURE LEGENDS

Supplementary figure 1. Representative images of expression of Ki-67 antigen (A,C) and β-catenin (B,D) of tumors cells from groups with only inoculation of CCR cell line without surgery (group 1) and inoculation of CCR cells on deportalized lobe and first step of ALPPS (group 2). Although proliferative index was similar in both groups (A, C), the expression of β-catenin differed. Thus, while in group 1 (B) expression was weak and with a membrane-pattern staining, in group 2 (D) many cells expressed a cytoplasmic/nuclear staining pattern.ABC anti-β-catenin stain. Scale bar: 100μm.

Supplementary figure 2. Representative images of COX-2 expression of Kupffer cells (KCs) in hepatic tissue from group with only inoculation of CCR cell line without surgery (group 1, A), inoculation of CCR cells on atrophic lobe and first step of ALPPS (group 2, B) and group with first step with ALPPS alone (group 3, C). While few KCs expressed COX-2 in group 1 (A, head arrow), all KCs in groups 2 (B) and 3 (C) expressed COX-2 (head arrows). ABC anti-COX2 stain. Scale bar: 100μm.

Supplementary figure 3. Representative images of immunophenotypical characterization of tumor-associated macrophages (TAMs) from group with only inoculation of CCR cell line without surgery (group 1, A-B) and inoculation of CCR cells on deportalized lobe and first step of ALPPS (group 2, C-D). While in group 1, there was very few COX-2 (A) or arginase-1 positive cells (B, head arrows), in group 2 there were low (C) COX-2 positive and high numbers of

arginase-1 positive TAMs (D, head arrows) expression. ABC anti-COX-2 (A,C) and arginase-1 (B,D) stains. Scale bar: 100μm.

Supplementary figure 4. Representative images of T-CD3$^+$ lymphocyte infiltrate (arrows) in hepatic tissue (A,C) and tumor (B,D) from group with only inoculation of CCR cell line without surgery (group 1) and inoculation of CCR cells on deportalized lobe and first step of ALPPS (group 2). While there is an increase of T-cells in hepatic tissue in group 2 (C, head arrows), there is also a decrease in intratumoral T-cell infiltrate (D). ABC anti-T-CD3 lymphocytes stain. Scale bar: 100μm.

Supplementary figure 5. Representative images of immunohistochemical expression of Hif1-α (A,C) and VEGF (B,D) on tumors from animals from group with only inoculation of CCR cell line without surgery (group 1) (A-B) and inoculation of CCR cells on atrophic lobe and first step of ALPPS (group 2) (C-D). In group 1, tumor cells were negative for HIF1-α (A), but were weakly positive for VEGF (B). In group 2, tumor cells were also negative for HIF1-α (C), were strongly positive for VEGF stain (D).ABC anti-HIF1-α (A,C) and VEGF (B,D) stains. Scale bar: 100μm.

SUPPLEMENTARY TABLE S1. Immunohistochemical expression of vasculogenic factors (HIF1-α and VEGF), β-catenin, and Kuppfer cells (KCs) COX-2 and Arginase-1 expression of hepatic and tumor tissues.

GROUP	LOBE	TISSUE	HIF1-α	VEGF	β-CATENIN	KCs COX-2	KCs ARGINASE-1
1	LEFT	*TUMOR*	-	+	MEMB (+)	-	-
		LIVER	-	-	MEMB	-	-
	RIGHT	*TUMOR*	-	+	MEMB (+)	-	-
		LIVER	-	-	MEMB	-	-
2	LEFT (FLR)	*LIVER*	+	++	MEMB	+++	-
	RIGHT (DEPORTALIZED)	*TUMOR*	-	+++	CIT/NUCL (++)	+	+++
3	LEFT (FLR)	*LIVER*	+	++	N/D	+++	-
	RIGHT (DEPORTALIZED)	*LIVER*	N/S	N/S	N/S	-	-
4	LEFT	*LIVER*	-	-	MEMB	-	-
	RIGHT	*LIVER*	-	-	MEMB	-	-

-: Negative (0-5%); +: low (5-15%); ++: moderate (15-30%); +++: high (>30%) number of positive cells. N/S: No viable tissue identified.

SUPPLEMENTARY FIGURE 1

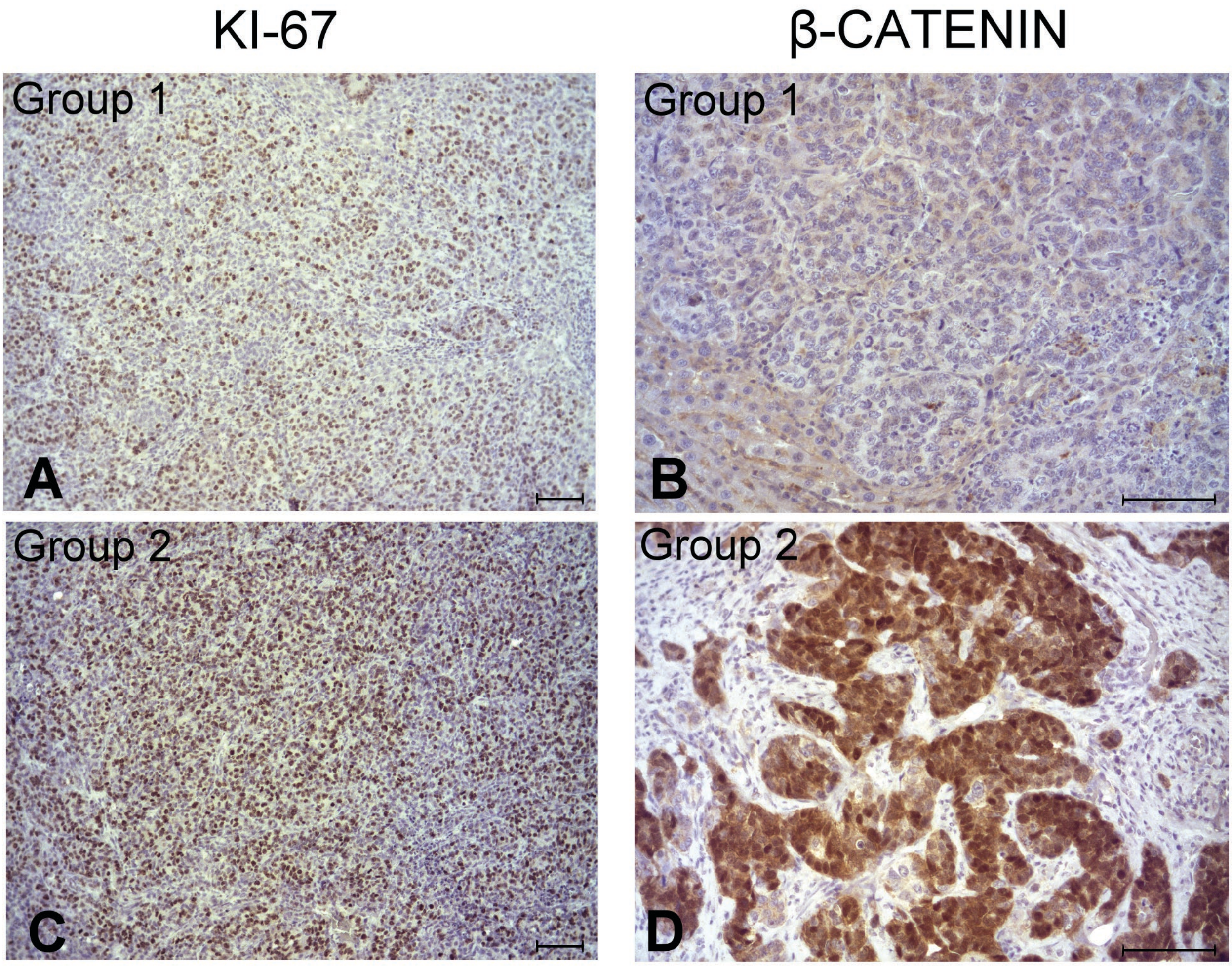

SUPPLEMENTARY FIGURE 2

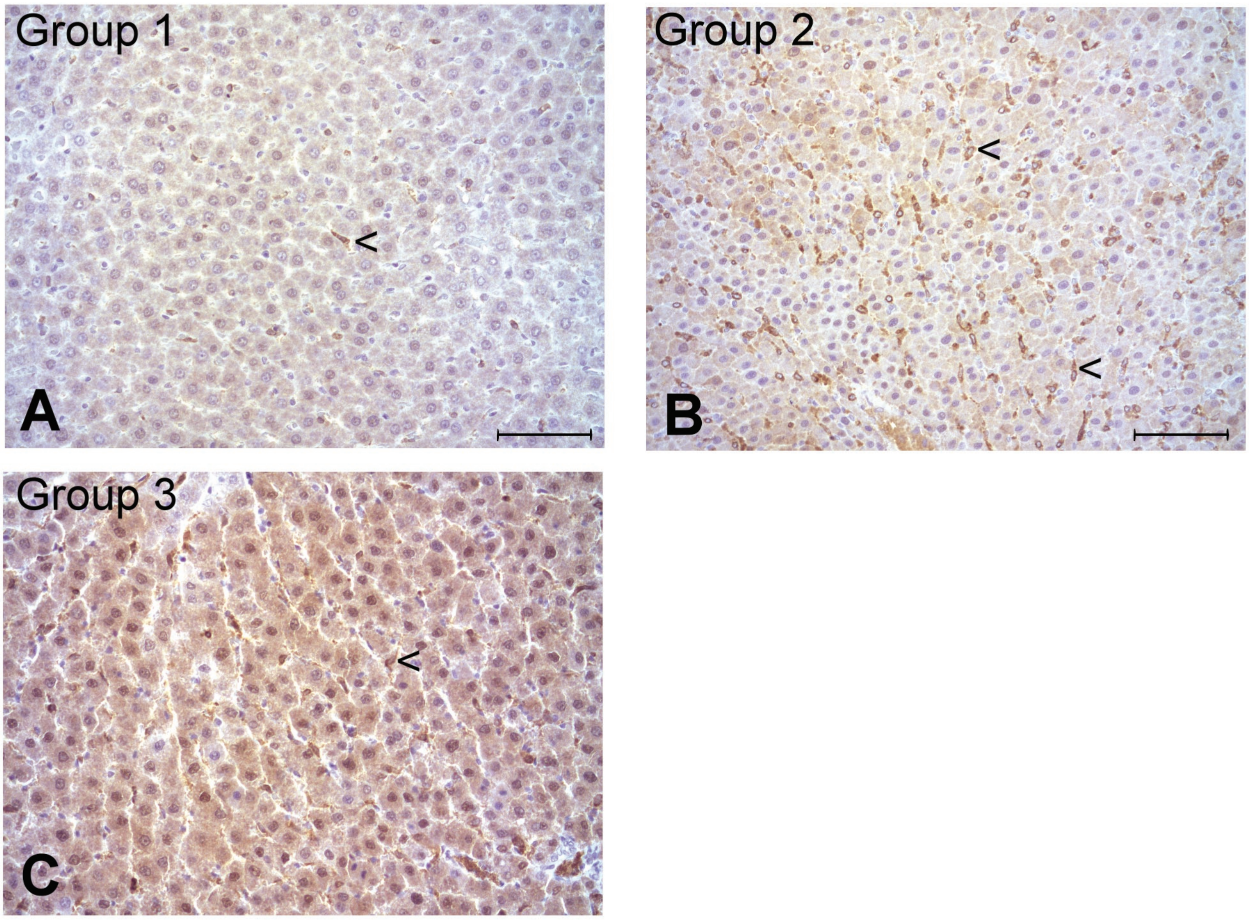

SUPPLEMENTARY FIGURE 3

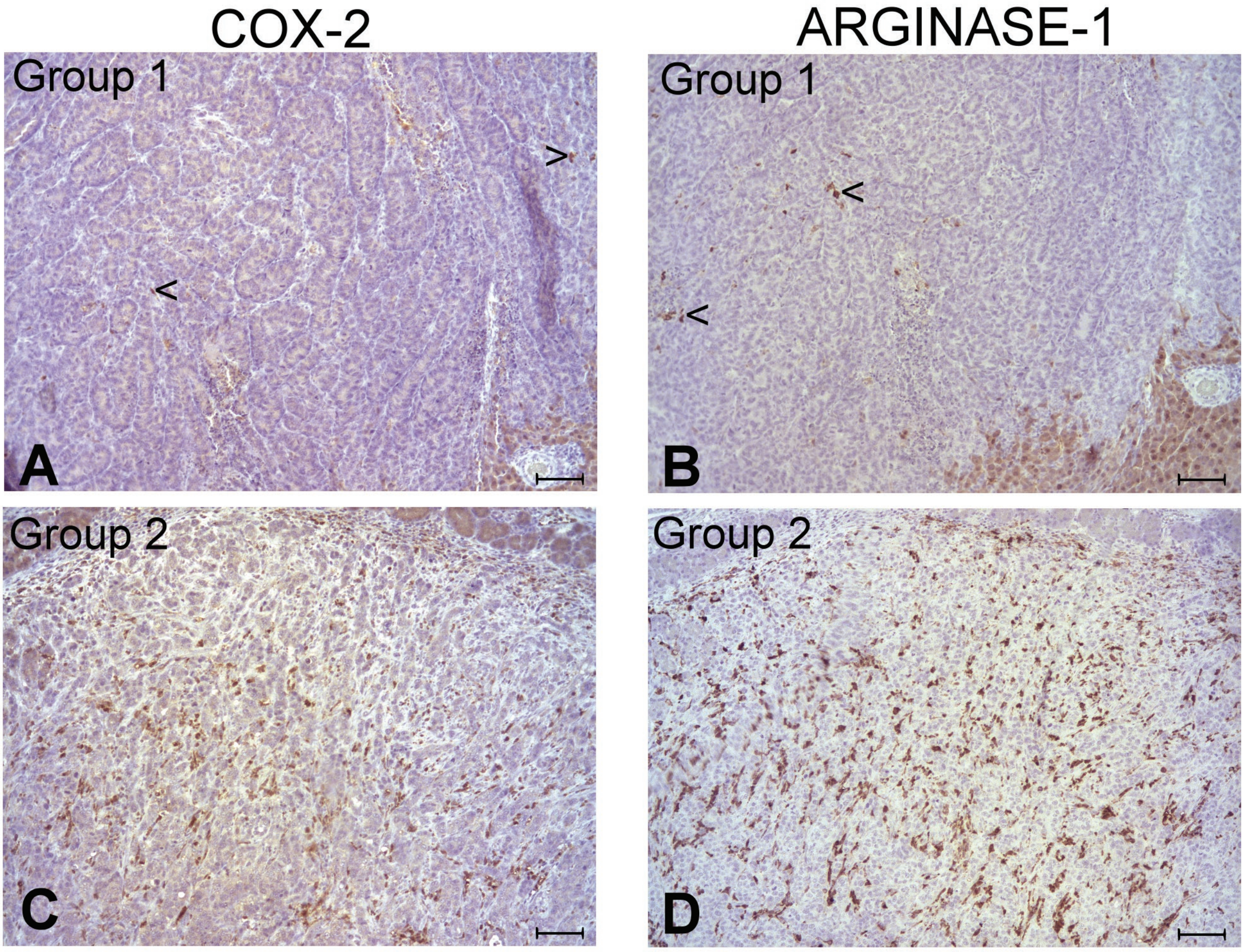

SUPPLEMENTARY FIGURE 4

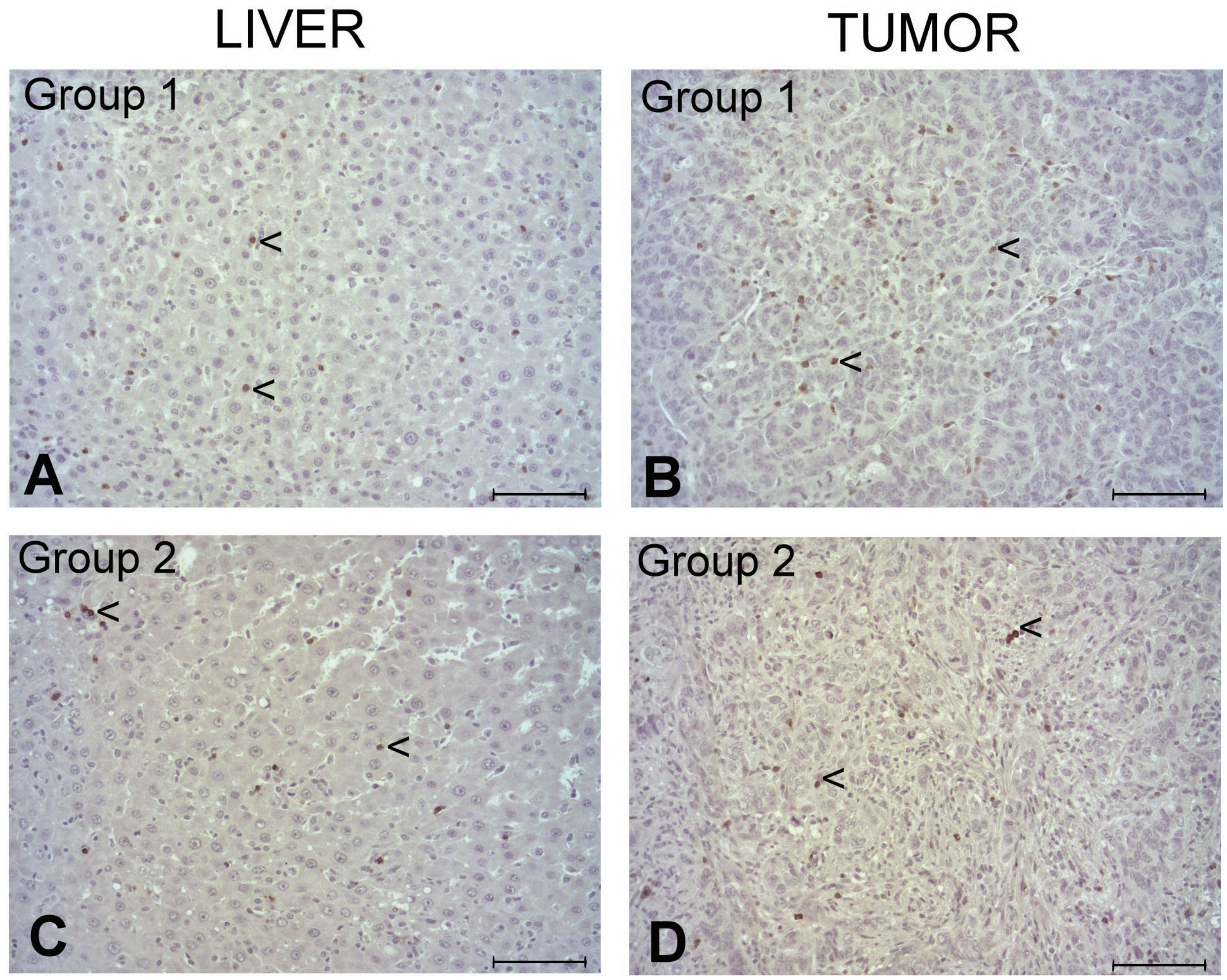

SUPPLEMENTARY FIGURE 5

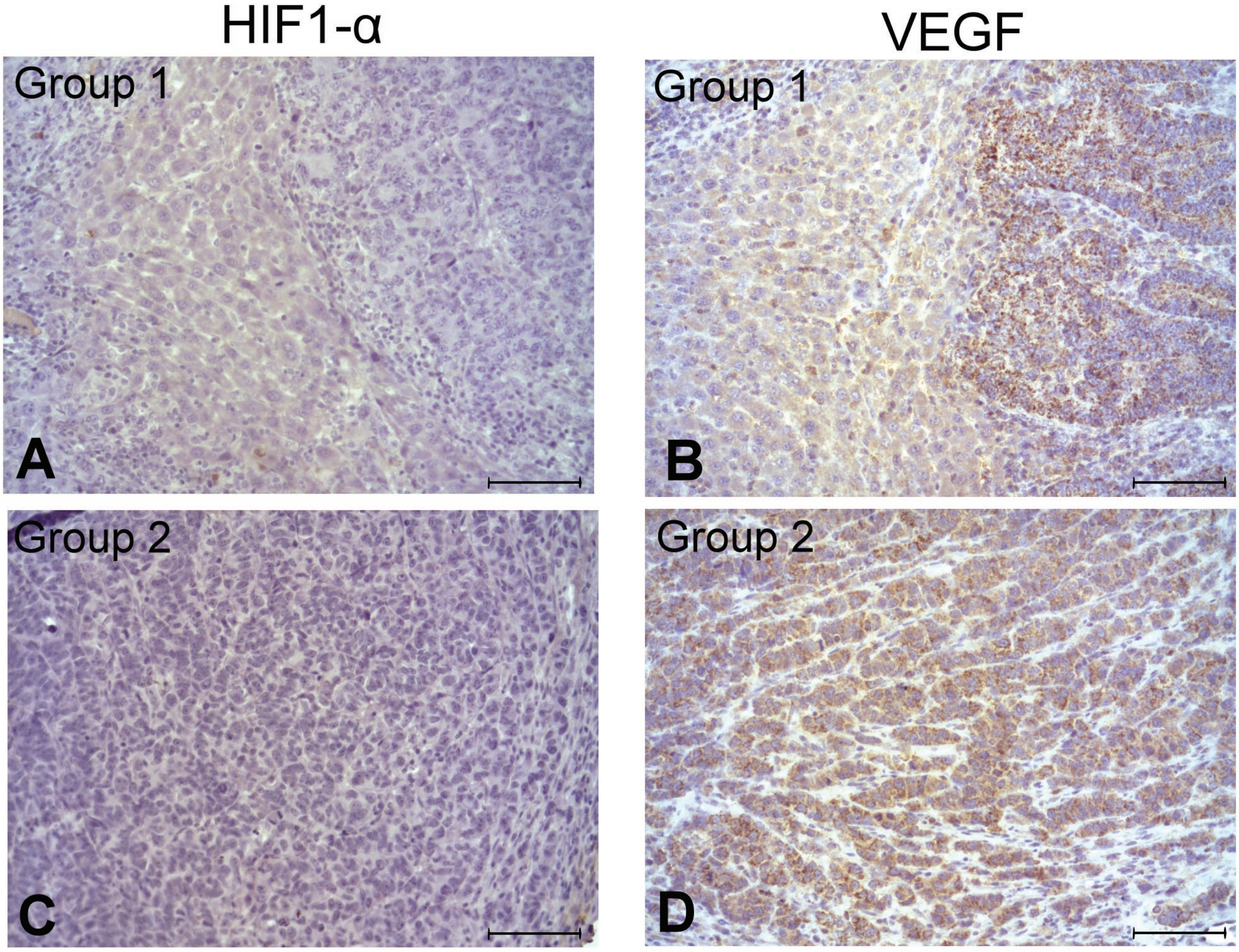

Supplement: Supplementary file 1 — Supplementary information [file 41598_2018_26082_MOESM1_ESM.pdf]
